# Diagnostic Accuracy of Short Tau Inversion Recovery as a Limited Protocol for Diagnosing Perianal Fistula

**DOI:** 10.7759/cureus.6398

**Published:** 2019-12-16

**Authors:** Naila Jabeen, Ruby Qureshi, Amjad Sattar, Musarat Baloch

**Affiliations:** 1 Radiology, Dow University of Health Sciences, Karachi, PAK; 2 Internal Medicine, Liaquat University of Medical and Health Sciences, Jamshoro, PAK

**Keywords:** short tau inversion recovery, sensitivity, specificity, perianal fistula

## Abstract

Introduction

Perianal fistula refers to abnormal communication between perianal skin and anal canal. Magnetic resonance imaging (MRI) and endoanal ultrasound have been used in the evaluation of perianal fistula. Endoanal ultrasound is a cost-effective but operator-dependent technique. MRI provides accurate details regarding anal canal anatomy and effectively identifies the fistulae. For evidence-based care, a cost-effective and an accurate imaging modality is required in a developing country. Therefore, the aim of this study was to determine the diagnostic accuracy of short tau inversion recovery (STIR) as a limited protocol MRI pelvis in diagnosing perianal fistula taking surgical findings as the gold standard.

Materials and methods

A retrospective review of MRI pelvis from 1^st^ February 2018 to 1^st^ July 2018 was undertaken. Patients of any age or gender suspected to have perianal fistula were included. One radiologist interpreted the complete MRI pelvis and the other radiologist only viewed axial and coronal STIR sequences as a limited protocol. Sensitivity, specificity, positive predictive value (PPV), negative predictive value (NPV), and diagnostic accuracy of axial and coronal STIR sequence were calculated taking surgical findings as the gold standard.

Results

In total, 150 patients were included in this study. The mean age of the patients was 43.20 ± 13.75 years. In total, 122 (81.3%) were males and 28 (18.7%) were females. Using STIR as a limited protocol, fistulae were found in 125 (83.3%) patients, whereas on surgery, the fistulae were found in 119 (79.3%) patients. Sensitivity, specificity, PPV, NPV, and diagnostic accuracy of STIR as limited protocol MRI pelvis in diagnosing perianal fistulae was found to be 96.6%, 67.7%, 92.0%, 84.0%, and 90.6%, respectively, taking surgical findings as the gold standard.

Conclusion

STIR has high sensitivity and diagnostic accuracy in diagnosing in the perianal fistula. Using STIR as a limited protocol in a developing country can help improving patient care by accurately diagnosing perianal fistulae. Moreover, it is recommended that further studies for identifying internal opening on STIR should also be carried out to improve patient care.

## Introduction

Perianal fistula usually refers to an abnormal communication existing between the perianal skin and the anal canal. It occurs due to obstruction in drainage of glands of the anal canal into the canal lumen and infection, therefore spreads to fatty tissues which provide little resistance to infection progression [1]. Crohn’s disease and malignancies are also an important cause of perianal fistula development [2-3]. Park’s classification is widely used to categorize the fistula [1]. Magnetic resonance imaging (MRI)-based classification system has also been developed by radiologists of St. James University Hospital [4]. It divides fistula into five grades. Grade 1 refers to simple linear intersphincteric fistula, grade 2 refers to intersphincteric fistula with secondary tract or abscess, grade 3 refers to transsphincteric fistula, grade 4 refers to transsphincteric fistula with secondary tract or abscess, and grade 5 refers to supralevator or translevator extension of the tract.

MRI and endoanal ultrasound have been used in the evaluation of perianal fistula [5-6]. Endoanal ultrasound is a cost-effective technique and almost comparable to examination under anesthesia. However, it is operator dependent and usually not much helpful if tracts or abscesses are located above the puborectalis muscle [7]. Recently, three-dimensional endoanal ultrasound has also been used for the evaluation of perianal fistulae with an AUC value of sensitivity being 0.97 and of specificity being 1.00 [8].

MRI provides accurate details regarding anal canal anatomy and effectively delineates fistulous tracts along with other findings such as abscesses or secondary tracts [9]. In MRI pelvis, T1 weighted, T2 weighted, short tau inversion recovery (STIR) and T1 post-contrast with fat suppression sequences are commonly used to evaluate fistula-in-ano [10]. Recently, the role of diffusion-weighted imaging (DWI) has also been evaluated [11]. However, MRI is an expensive modality and is time-consuming to perform. STIR sequences do not require contrast injections and are a part of a complete MRI protocol for perianal fistulae. A recent study has shown that the STIR sequence is highly sensitive for diagnosing perianal fistula [12].

For evidence-based care, a cost-effective and accurate imaging modality is required in a developing country like Pakistan where many people cannot afford quality care. To the best of our knowledge, no published local data exists on the current topic in our population. Therefore, the aim of this study was to determine the diagnostic accuracy of STIR as a limited protocol MRI pelvis in diagnosing perianal fistula taking surgical findings as the gold standard.

## Materials and methods

A retrospective review of MRI pelvis from 1st February 2018 to 1st July 2018 was undertaken. Patients of any age or gender suspected to have perianal fistula were included. MRI pelvis performed for any other indication was excluded. Two radiologists blinded to the findings of each other interpreted the MRI. One radiologist interpreted the complete MRI pelvis and the other radiologist only viewed axial and coronal STIR sequences as a limited protocol. Mean and standard deviation (SD) was calculated for quantitative variables such as age. The frequency and percentages were calculated for qualitative variables like gender, presence of fistula on STIR and surgery. Sensitivity, specificity, positive predictive value (PPV), negative predictive value (NPV) and diagnostic accuracy of STIR as a limited protocol was calculated taking surgical findings as the gold standard. Effect modifiers such as age and gender were stratified and post-stratification sensitivity, specificity, PPV, NPV, and diagnostic accuracy was ×calculated.

## Results

The mean age of the patients was 43.20 ± 13.75 years. In total, 89 (59.3%) patients presented with ≤45 years of age. In total, 122 (81.3%) were males and 28 (18.7%) were females. The baseline characteristics of the patients are summarized in Table 1.

**Table 1:** Baseline characteristics of the patients

Baseline characteristics of the patients (n = 150)		
	n	%
Age, years	43.20 ±13.75^ǂ^	
≤45 years	89	59.3
>45 years	61	40.7
Gender		
Males	122	81.3
Females	28	18.7
^ǂ^mean±SD, n: number		

Fistulae on limited protocol MRI were found in 125 (83.3%) patients, whereas on surgery, the fistulae were found in 119 (79.3%) patients (Table 2).

**Table 2:** Limited protocol MRI and surgical findings MRI, magnetic resonance imaging

Limited protocol MRI and surgical findings (n = 150)			
Limited protocol MRI findings	Surgical findings		Total
	Positive	Negative	
Positive	115	10	125
Negative	4	21	25
Total	119	31	150

According to St. James classification of fistula on MRI, the most common type was grade I followed by grade III (Table 3).

**Table 3:** St. James classification of fistulae according to MRI MRI, magnetic resonance imaging

St. James classification of fistulae according to MRI		
	n	%
Grade I	70	46.7
Grade II	18	12.0
Grade III	30	20.0
Grade IV	7	4.7
Grade V	0	0.0

Sensitivity, specificity, PPV, NPV, and diagnostic accuracy of STIR as limited protocol MRI pelvis in diagnosing perianal fistulae was found to be 96.6%, 67.7%, 92.0%, 84.0%, and 90.6%, respectively, taking surgical findings as the gold standard.

## Discussion

Perianal fistula is an inflammatory disease process occurring as a result of an abnormal connection between the anal canal and the perineum skin. It presents with perianal pain and discharge and impairs the quality of life of patients. The adequate management of perianal fistulae requires good preoperative planning such as diagnosis and extent of fistula and the location of internal opening [13]. Perianal fistula can be successfully treated by surgery. For complete cure, it is essential to remove all the infective areas associated with the fistulous tract. MRI is currently the standard imaging technique for the accurate evaluation of perianal fistulae. The role of CT fistulography has also been studied but its use is not widespread. In the preoperative planning, it may supplement MRI [14]. Preoperative MRI can help in reducing the recurrence and is also helpful in the evaluation of complex fistulous disease process [15]. MRI sequences that are usually performed for perianal fistula assessment include plain and post-contrast T1-weighted and T2-weighted sequences [10]. STIR sequences have also been used [12]. However, there are controversial details related to the use of gadolinium-based contrast agents [16].

This study was an attempt to evaluate the use of axial and coronal STIR sequences together as a limited protocol for evaluation of perianal fistula in a country where the majority of the population belongs to the socioeconomic status of lower class or lower middle class. In evaluating these, we found that the findings were visible without the loss of information. Moreover, a high sensitivity, moderate specificity, and high diagnostic accuracy of STIR were demonstrated in diagnosing perianal fistula taking surgical findings as the gold standard.

**Figure 1:**
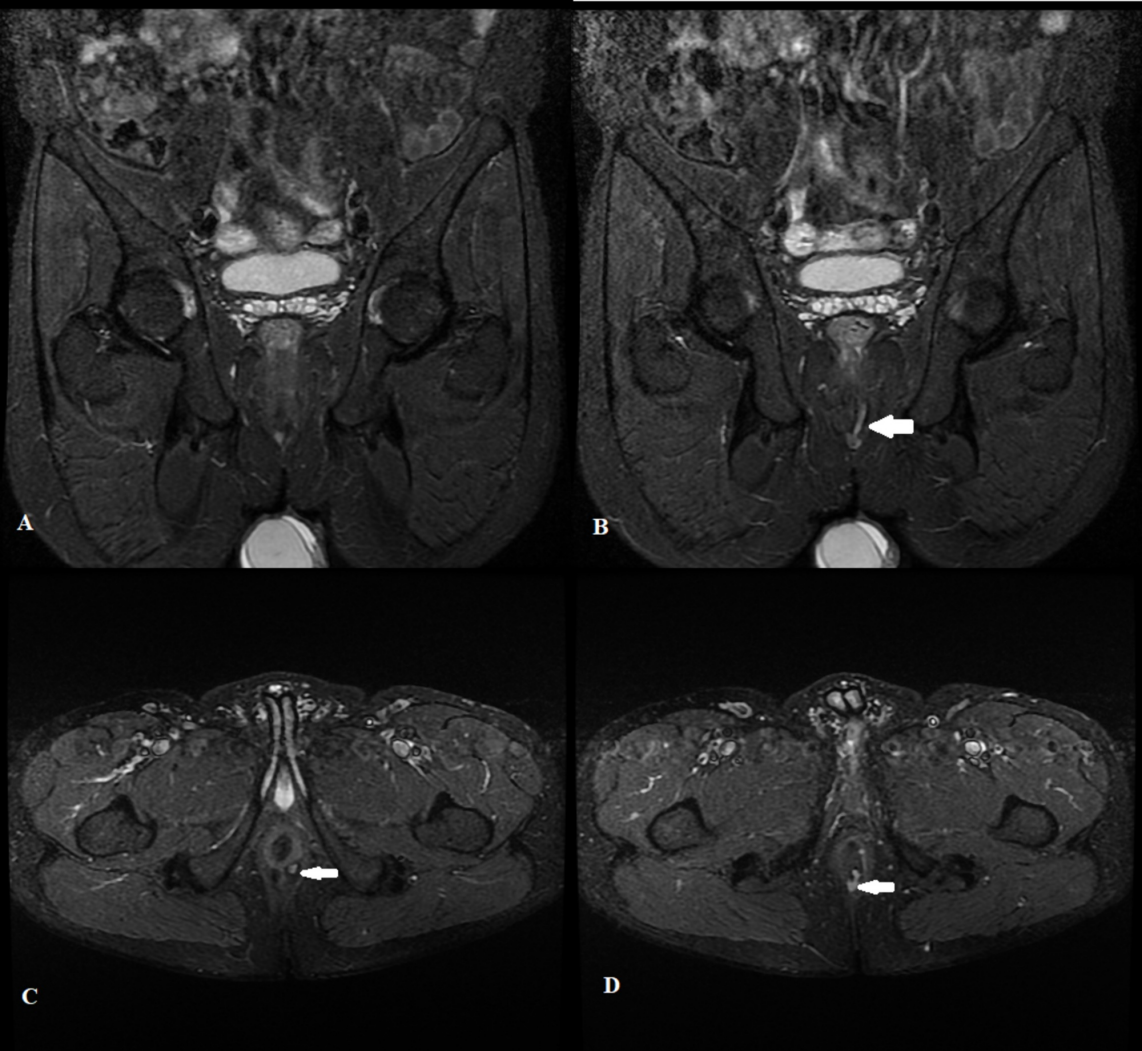
Short tau inversion recovery sequence showing perianal fistula Coronal (A and B) and Axial (C and D) short tau inversion recovery images of a 37-year-old male demonstrating a linear intersphincteric perianal fistula (white arrow) with its internal opening at 5'o clock position

Our study showed a high sensitivity of the STIR sequence in diagnosing perianal fistula. This sensitivity is almost comparable to the one reported by another study [17]. STIR sequences are commonly used for the anatomical regions that have a high fat content where the important aspect of inversion recovery can be applied [18]. The visualization of a fistulous tract depends upon their content as well as the degree of inflammation. STIR suppresses the signals of adjacent fat and provides a good contrast to identify the fistula easily. In the active disease phase, the fistula usually contains granulation tissue with fluid and is usually seen as a linear tract of high signal on STIR (Figure 1).

Moreover, on STIR sequences, it is easy to identify fistula within the external sphincter because the sphincter shows moderately low signals on STIR and its walls contrast with fat of the ischioanal fossa.

The specificity of STIR for perianal fistula in our study was lower as compared to the one reported by the other study [17]. However, another study utilizing the endoanal coil demonstrated the specificity that was comparable to our study [19]. According to another study, STIR and fat-suppressed T2-weighted images are sufficient to diagnose perianal fistula; however, classification on STIR is better due to the fact that delineation of pelvic floor musculature is easier [18]. Moreover, the authors of the study also suggest that T1-weighted images have generally no contribution in diagnosing perianal fistula [18]. The positive and negative predictive values of STIR were also reported high in our study.

Our study was not without certain limitations. Firstly, a major limitation of our study was that we did not determine the sensitivity and specificity of STIR for diagnosing internal opening. Secondly, our study was done on the small sample size and was a single-institution study. Another limitation of our study was that the interobserver and intraobserver agreement was not calculated. Observer agreement forms an important part of radiological imaging and it can help in a better understanding of the imaging technique. Therefore, it is recommended that multicentric studies on a larger sample size should be carried out in our population to evaluate the interobserver agreement and diagnostic accuracy including sensitivity and specificity for identifying the internal opening of perianal fistula.

## Conclusions

STIR has high sensitivity and diagnostic accuracy in diagnosing in the perianal fistula. Utilizing this sequence as a limited protocol in a developing country can help improve patient care by accurately diagnosing perianal fistulae. Moreover, it is recommended that further studies for identifying internal opening on STIR should also be carried out to improve patient care.
